# JUNTOS Radio: a podcast created in collaboration with Spanish-speaking healthcare providers, Juntos Center for Advancing Latino Health, and a medical librarian

**DOI:** 10.5195/jmla.2023.1653

**Published:** 2023-10-02

**Authors:** Brenda M. Linares, Mariana Ramirez

**Affiliations:** 1 blinares@kumc.edu, University of Kansas Medical Center, Kansas City, KS.; 2 mramirez3@kumc.edu, University of Kansas Medical Center, Kansas City, KS.

**Keywords:** Outreach, podcast, Spanish, Collaboration, Consumer Health, community engagement, Latinx, COVID-19

## Abstract

Spanish speaking healthcare providers, JUNTOS Center for Advancing Latino Health, and a medical librarian partnered to create a podcast on essential health topics relevant to the Latinx community. The podcasts were recorded in Spanish and included Spanish supplementary consumer health information from credible resources such as MedlinePlus en Espanol. The podcasts covered important topics about COVID-19 such as vaccines, clinical trials, and social distancing. It also includes other relevant topics that are affecting the Latinx community.

## INTRODUCTION

A medical librarian partnered with the director of JUNTOS Center for Advancing Latino Health and Spanish-speaking health care providers to provide credible health information to the Latinx community through recorded Spanish podcasts.

The idea for the podcast project started in 2019 when a medical librarian, Brenda Linares, at the University of Kansas Medical Center A.R. Dykes Library and the director of the JUNTOS Center for Advancing Latino Health (https://juntosks.org/), Mariana Ramirez, met to brainstorm ideas on collaborative opportunities to reach the Latinx community to promote health literacy and reduce misinformation. Mariana had the idea of creating Spanish-language podcasts, and the medical librarian added that relevant Spanish-language consumer health information could be incorporated with each corresponding topic. There is a shortage of Spanish-speaking health care providers and there is a lot of mistrust and misinformation in the Latinx community, therefore, this idea was a good way to reach out and educate this community [1]. Funding from the Network of the National Library of Medicine (NNLM) and All of Us research was used to support this project. The project aim was to increase access to reliable health information for Spanish-speaking Latinos in Kansas and other regions. JUNTOS wanted to share health information through a culturally and linguistically tailored podcast that would develop a sense of trust among the community, health professionals, and librarians. That is when the idea to develop and produce quality, evidence-based, smartphone-friendly content tailored to the linguistic, literacy, and cultural needs of medically underserved Latino subgroups came to be.

## PHASE 1

During the first phase in 2019, the JUNTOS Center along with a librarian conducted initial literature reviews and worked with an online outreach specialist consultant to develop a multiplatform community outreach plan. The project team developed a list of Spanish-speaking health care professionals in different fields including chronic disease management, nutrition, mental health, and Alzheimer's disease that could participate in the podcast. The team worked with a community outreach strategy specialist with expertise in Latino markets on strategies to raise awareness of JUNTOS on Facebook and other social media platforms. YouTube and Facebook were free platforms, Podbean had a free version and a monthly fee subscription depending on the storage needed.

## PHASE 2

During the second phase of the project, the team recorded episodes released to the public. The episodes included: 1) interviews with Spanish-speaking health professionals, 2) updates on relevant health research in plain language, and 3) storytelling from community members sharing health-related experiences and resources. We conducted focus groups and surveys to assess the acceptability of the episodes and participants' knowledge of the National Library of Medicine (NLM) Consumer Health Information resources before and after listening to the episodes. Seventy-six percent of the participants stated that they knew more about health topics after listening to the Podcast and that they would visit NLM resources.

Funding covered equipment including microphones, external drives, a video camera, headphones, transcript services, and Wildstyle Media Consulting. When the project started in 2020, the team had a list of topics planned out including health literacy and diabetes. But as the COVID-19 pandemic started, the group changed plans, and recordings were done via Zoom. In-person focus groups were moved into a virtual setting. The project team focused on topics relevant to the pandemic such as social distancing, clinical trials, vaccine hesitancy, and keeping active during the pandemic. Once these podcasts were recorded, they were uploaded to Podbean, the YouTube Channel, Facebook, and Apple podcast making them findable by the general public and free to access in a variety of popular ways.

## CONCLUSION

This project has continued to provide new podcasts about relevant topics for the Latinx community. The team hopes that community leaders and promotoras find out about it, listen to the episodes, and use it with the Latinx communities they work with across the country. The podcast has credible and relevant topics that are helpful and can reduce misinformation (find out more and listen to the podcast at https://juntosradio.podbean.com/).

Developed resources reported in this presentation are supported by the National Library of Medicine (NLM), National Institutes of Health (NIH) under cooperative agreement number UG4LM012344. The content is solely the responsibility of the authors and does not necessarily represent the official views of the National Institutes of Health.
